# Multiple approaches to understanding the taxonomic status of an enigmatic new scorpion species of the genus *Tityus* (Buthidae) from the biogeographic island of Paraje Tres Cerros (Argentina)

**DOI:** 10.1371/journal.pone.0181337

**Published:** 2017-07-26

**Authors:** Andrés A. Ojanguren-Affilastro, Renzo S. Adilardi, Rodrigo Cajade, Martín J. Ramírez, F. Sara Ceccarelli, Liliana M. Mola

**Affiliations:** 1 Museo Argentino de Ciencias Naturales “Bernardino Rivadavia”, Buenos Aires, Argentina; 2 Laboratorio de Citogenética y Evolución—Departamento de Ecología, Genética y Evolución, IEGEBA (CONICET-UBA), Facultad de Ciencias Exactas y Naturales, Universidad de Buenos Aires, Buenos Aires, Argentina; 3 Laboratorio de Herpetología, Departamento de Biología, Facultad de Ciencias Exactas y Naturales y Agrimensura, Universidad Nacional del Nordeste, Corrientes, Argentina; 4 Departamento de Biología de la Conservación, Centro de Investigación Científica y de Educación Superior de Ensenada, Baja California, México; Natural Resources Canada, CANADA

## Abstract

*Tityus curupi* n. sp., belonging to the *bolivianus* complex, is described from the biogeographically distinct area of Paraje Tres Cerros in north-eastern Argentina. We also present a molecular species delimitation analysis between *Tityus curupi* n. sp. and its sister species *Tityus uruguayensis* Borelli 1901 to confirm species integrity. Furthermore, a cytogenetic analysis is presented for these two species which contain different multivalent associations in meiosis, as a consequence of chromosome rearrangements, and the highest chromosome numbers in the genus.

## Introduction

The scorpion family Buthidae occurs on all continents except Antarctica, and is present in most of the tropical and temperate areas of the world. *Tityus* is the most diverse genus of Buthidae, with more than 200 described species. It occurs in some of the Antillean islands, Central and South America, and is especially diversified in tropical areas. In southern and temperate South America the diversity of *Tityus* species is comparatively marginal, with only few species occurring in the area [1]. Due to its diversity, this genus has been separated in several groups and complexes of species (both terms will be used as synonyms from now on), which could correspond to monophyletic groups [2, 3, 4, 5, 6, 7, 8, 9]. It has also been separated in different subgenera [10] but unfortunately with no phylogenetic analysis to support them. The taxonomy of *Tityus* is still confusing since most of the contributions in the family are isolated morphological descriptions, usually following old standards, which makes a revision of the genus mandatory.

Our research group has recently published the first dated molecular phylogeny of the southernmost neotropical buthids [11], which provided a first step for the revision of genus *Tityus* in the area. Our results do not support the subgeneric division proposed by Lourenço [10], nor the validity of some of the groups of species of the area, since subgenus *Tityus*, as currently defined, appears as paraphyletic, and group *confluens* as a synonym of group *trivittatus* [11]. Groups *bolivianus*, *clathrattus*, and *trivittatus*, on the other hand, appear as well supported, and most probably correspond to monophyletic groups.

**Fig 1:**
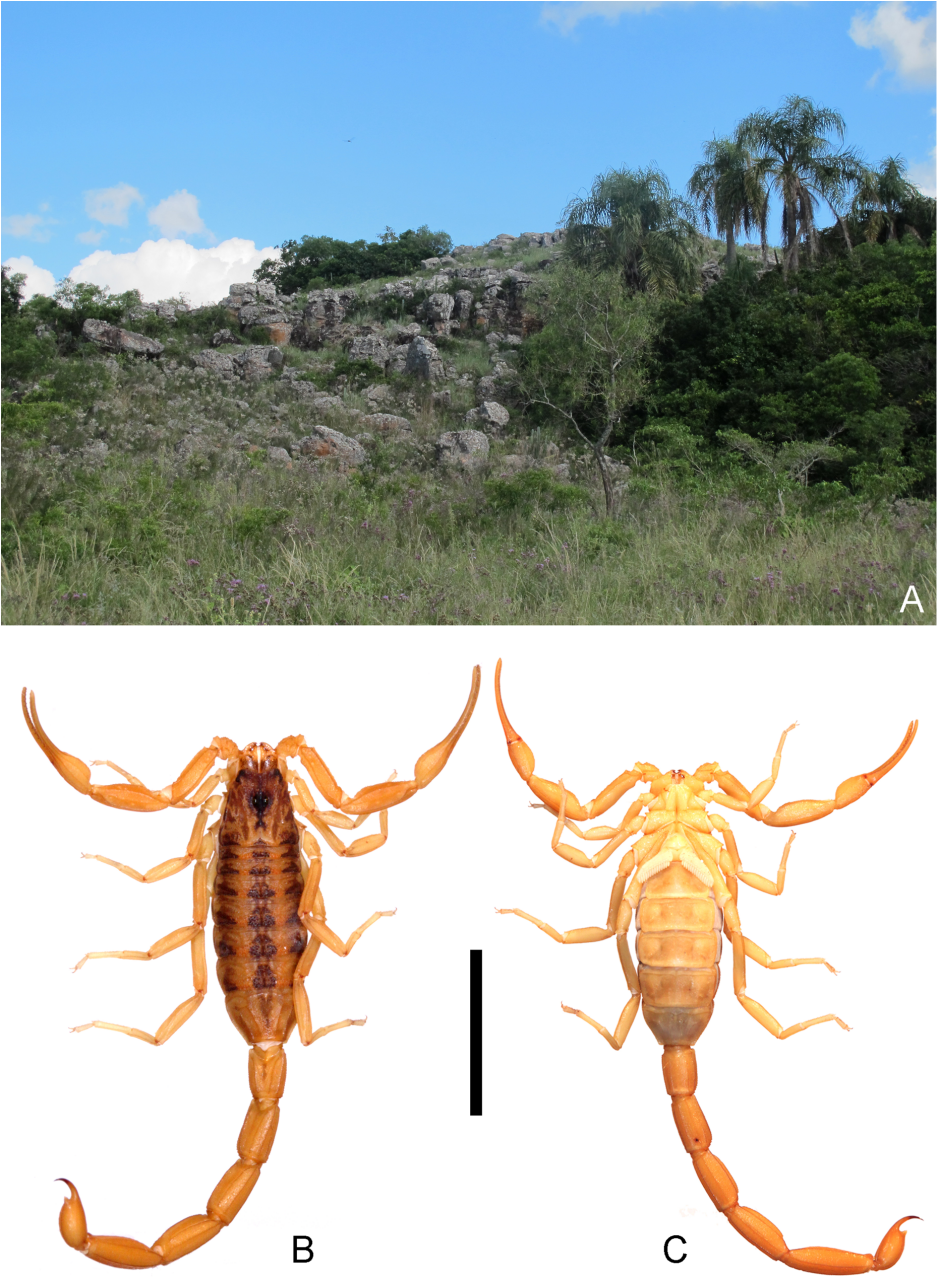
Habitat and habitus of *Tityus curupi* n. sp. A. Cerro Capará, Paraje Tres Cerros, Corrientes, Argentina, type locality of *Tityus curupi* n. sp. B, C. *Tityus curupi* n. sp. habitus female. B. Dorsal aspect; C. Ventral aspect. Scale bar = 1cm.

**Fig 2:**
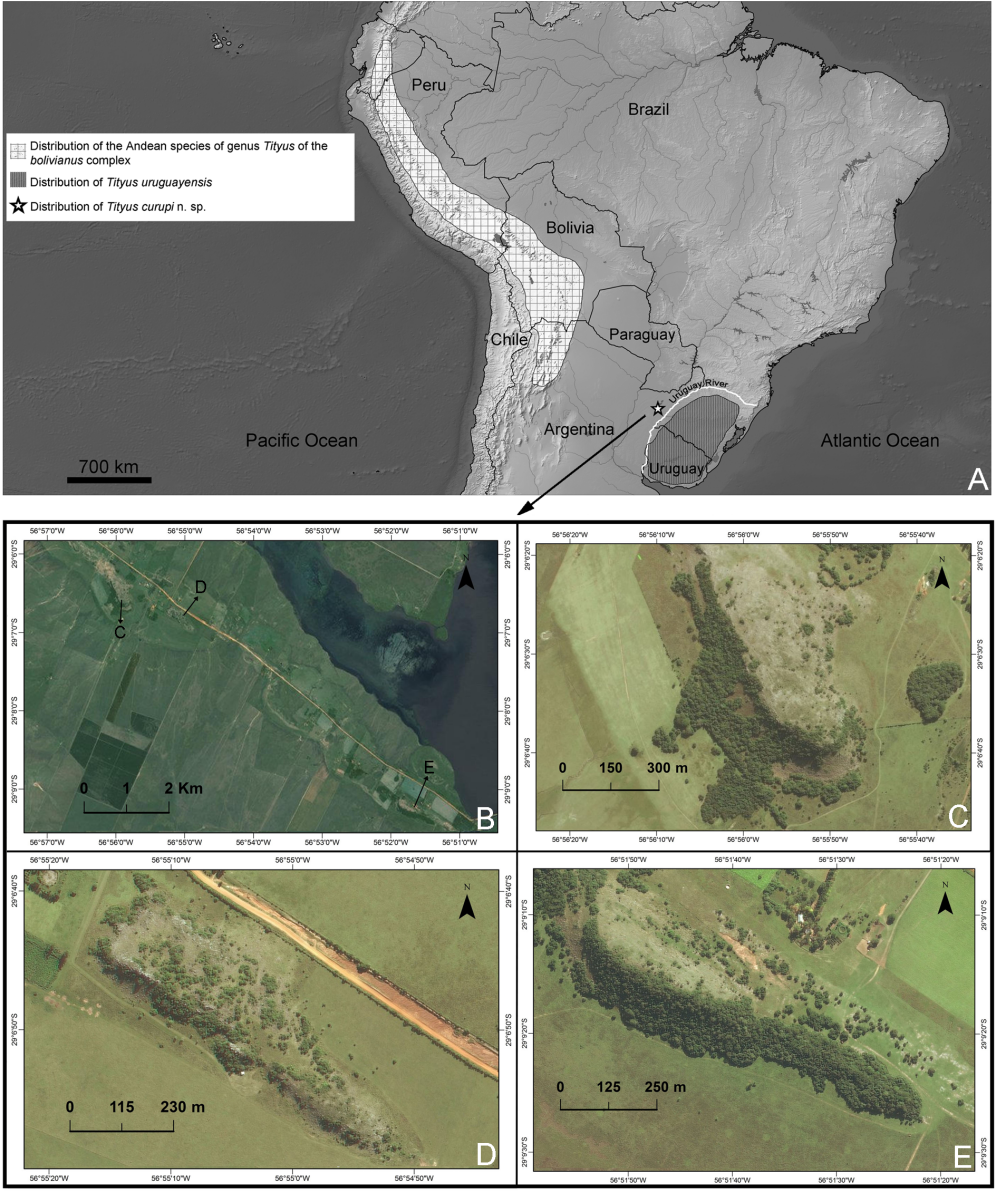
Ditribution map of *Tityus curupi* n. sp. A. Distribution map of the species of *Tityus* of the *bolivianus* complex including the type locality of *Tityus curupi* n. sp. Uruguay River is also indicated with a white line. B-E. B. Detail of Paraje Tres Cerros, Corrientes province, Argentina, type locality of *Tityus curupi* n. sp. Arrows indicate the exact position of the three hills of the area: C–E. Details of hills Nazareno, Chico and Capará respectively.

In the last five years, part of our research group has been carrying out several collection campaigns to the hills of Paraje Tres Cerros. This is an isolated low altitude hilly area of subtropical western Argentina composed of three rocky isolated hills of 150–180 m asl of quartz sandstone [12, 13, 14], corresponding to the Botucatu stratigraphic formation (Figs 1A and 2) [15]. Two hypotheses are currently being considered to explain the emergence of these hills: 1) a relatively early emergence, about 10–5 million years ago (Ma), related to the final rapid uplift of the Andes [16, 17], or 2) a much older Gondwanic origin. Due to their different environmental conditions with respect to their surrounding areas, these hills have been a “biogeographic island” for a long time, and they host several endemic species of plants [18, 19, 20] and small vertebrates [13, 14]. As a result of these campaigns our research group collected several new endemic species of epigean fauna, including an enigmatic new scorpion species: *Tityus curupi* n. sp. that we describe herein (Fig 1B and 1C). This species belongs to the *bolivianus* complex; species of this complex, or group of species, are slender and medium sized; their mesosoma presents three dorsal longitudinal dark stripes (which are less evident in some species); the basal median pectinal lamella of females is always lobular and much larger than in males [7]; the hemispermatophore lacks the median lobe, and the external lobe is fused dorsally to the trunk by a thin lamina [1]; distal denticles of DL carinae of metasomal segments II-IV are slightly spiniform, and metasomal segments of males are usually more elongated than in females [7]. This species complex presents a peculiar disjunct distribution, with 14 described species occurring at intermediate altitudes in the Andes from Ecuador to Argentina and only one known species occurring in plains of southern Brazil and Uruguay, *Tityus uruguayensis* Borelli 1901(with one introduced population in Argentina) [7, 1, 11]. This species complex was thought to be absent from the intermediate areas of Chaco, Espinal and Paranaense Forests; therefore the presence of this new species in the isolated hills of Paraje Tres Cerros is particularly intriguing because it represents the first record of this group in the lowlands west of the Uruguay River.

The morphology of *T*. *curupi* n. sp. is very similar to that of its closest relative, *T*. *uruguayensis*. Due to this, and because of the confusing situation of *Tityus* taxonomy, we decided to deal with the description of these species from different points of view: a morphologic description, a molecular species delimitation analysis between *T*. *curupi* n. sp. and its sister species *T*. *uruguayensis*, and a detailed cytogenetic analysis of both species, allowing us to compare their karyotypes, meiotic configurations and ribosomal DNA (rDNA) and (TTAGG)_*n*_ telomeric repeats localization.

The genus *Tityus* presents several characteristics that make it of particular interest for cytogenetic studies. This genus, as all the Buthidae scorpions, has holokinetic chromosomes (i.e. chromosomes without a primary constriction) and lacks meiotic recombination in males. *Tityus* also shows a wide interspecific variation in chromosome number (from 2n = 5 to 2n = 27), as well as many cases of intraspecific variation. Another unusual cytogenetic feature present in 10 of 12 species of *Tityus* with cytogenetically studied males is the high incidence of chromosome rearrangements in heterozygous state, giving rise to multivalent associations at meiosis I [21, 22, 23, 24, 25, 26, 27, 28, 29]. Up to date, the only cytogenetic data from a species of *bolivianus* the complex belongs to *Tityus argentinus* Borelli 1899, which presents 2n = 10 with all bivalents at meiosis I and 2n = 9 with a bivalent and a heptavalent [30].

Molecular species delimitation analyses have proved to be a reliable tool to deal with closely related species of scorpions with difficult taxonomy, as those of genus *Brachistosternus* [31, 32]. This is the first time that this technique is applied in species of genus *Tityus*.

## Materials and methods

### Taxon sampling

Most specimens were manually collected by the authors at night using UV lamps, or during the day under stones or logs, or in the base of large grasses. Permits for legal collection in Reserva Natural Privada Paraje Tres Cerros were obtained in the “Departamento de Fauna Silvestre, Dirección de Recursos Naturales, Ministerio de Producción, Trabajo y Turismo de la provincia de Corrientes”, Argentina. This reserve is placed in private areas of “Estancia Higuera-Cue”, and “Estancia Las Marias”; in each case the owner of the land gave permission for our survey. Permits for Legal Collection in El Palmar National Park, were obtained in the Argentinean National Park Administration. All specimens are deposited in the Museo Argentino de Ciencias Naturales Arachnological collection (MACN-Ar), Buenos Aires, Argentina. Sequences are deposited in GenBank (Table A in S1 Table).

### Taxonomy

Descriptive terminology follows [1, 33, 34] for hemispermatophores; [35] for trichobothria; [36] for metasomal carinae, abbreviated as follows: DL: dorsolateral; LIM: lateral inframedian; LSM: lateral supramedian; LM: lateral median; VSM: ventral submedian; VL: ventrolateral; VM: ventromedian; and [37] for pedipalp carinae, abbreviated as follows: DI: dorsal internal; DE: dorsal external; VI: ventral internal; VE: ventral external; D: digital; E: external; IM: internomedian; EM: externomedian; V: ventral; VM: ventral median; DM: dorsal marginal; DS: dorsal secondary. Measurements, taken using an ocular micrometer, are expressed in mm.Digital images of pigmentation pattern and habitus were taken under visible light, images of external morphology under UV light, using a digital camera (Leica DFC290 or Nikon DS-Fi1) attached to a stereomicroscope (Leica M165C or Nikon SMZ1500), and the focal planes fused with Helicon Focus 3.10.3 (http://www.heliconsoft.com/heliconsoft-products/helicon-focus/). Point locality records were georeferenced in the field with portable Global Positioning System devices (Garmin® GPS II Plus, Etrex, Etrex Vista and Etrex Vista C) or retroactively using the GeoNet Names Server (http://geonames.nga.mil/gns/html/). A distribution map was generated using the web site www.simplemappr.net. Images made in World Imagery - ArcGIS. Sources: USGS (United State Geological Service) and IGN (Instituto Geográfico Nacional), Argentina.

In this contribution we use previous names of species groups and complexes for the genus *Tityus*, but to simplify the reading we generally refer to them as “complexes”.

### Molecular species delimitation

To test the validity of *T*. *curupi* n. sp. as a different species from *T*. *uruguayensis*, Bayes Factor species delimitation (BFD) [38] was applied to a dataset with six *T*. *uruguayensis*, six specimens belonging to *T*. *curupi* n. sp. and ten outgroup taxa, using three gene fragments: 491 base-pairs (bp) of the D3 region of the nuclear large-subunit ribosomal RNA (28S rDNA) gene, ca. 330 bp of the mitochondrial large-subunit ribosomal RNA (16S rDNA), and 654 bp of the mitochondrial Cytochrome c Oxidase Subunit I (COI) gene. The sequences from the outgroup taxa plus one sequence from a *T*. *uruguayensis* and one *T*. *curupí* n. sp. were obtained from a previous study [11], whereas the remaining sequences (five *T*. *uruguayensis* and five *T*. *curupí* n. sp.) were newly generated for this study. DNA extraction, amplification, sequencing and sequence alignment were carried out following the protocols outlined in [11]. Primers used for PCR amplification of gene fragments for the Bayes Factor species delimitation analyses in Table B in S1 Table. The ten outgroup taxa comprised five *Tityus*: *Tityus argentinus* Borelli 1899, *Tityus bahiensis* (Perty 1833) (type species of the genus), *Tityus confluens* Borelli 1899, *Tityus paraguayensis* Kraepelin 1895 and *Tityus trivittatus* Kraepelin 1898, four *Zabius*: *Zabius birabeni* Mello-Leitão, *Zabius fuscus* (Thorell 1876) (type species of the genus), *Zabius* sp1 and *Zabius* sp2, and one *Ananteris*: *Ananteris balzanii* Thorell 1891 (type species of the genus) (list of studied material in S1 File). The specimens of *T*. *uruguayensis* used for molecular studies belong to the only known population of the species in Argentina, from an area around the ruins of an old human settlement in El Palmar National Park. We have extensively surveyed nearby similar areas, and we could not find this species. Therefore due to its characteristics, we consider that this population of *T*. *uruguayensis* has an anthropic origin and that this species is not part of the native epigean fauna of the west side of the Uruguay River basin [1].

To get a first idea of genetic distance between *T*. *uruguayensis* and one *T*.*curupi* for the three markers used here, we calculated their “net between group mean distances” in MEGA7 [39]. Next, BFD was carried out on the complete alignments. BFD takes incongruence between gene trees and species trees [40] into account by applying the multispecies coalescence analysis. For this study, two separate species-assignment hypotheses were tested using the coalescent species tree algorithm *BEAST [41] in BEAST v. 1.8.1 [42], while estimating the marginal likelihood (MLE) by path-sampling (PS), [43] and stepping-stone sampling (SS), [44]. In one hypothesis, the individuals belonging to the undescribed species were considered as conspecific to *T*. *uruguayensis*, while in the other hypothesis they were considered as a separate, new species. For the coalescent species tree analyses, molecular clock rates were estimated, nucleotide substitution models were simplified to HKY [45] for all partitions to avoid over-parametrization, the prior for the species tree was set as a Birth-Death process and the piecewise linear and constant root was used for the population size model. Lognormal priors with initial values of 1, mean values of 0.01 and 1 standard deviation were set as priors for mean uncorrelated lognormal clock, population hyper-parameter and Yule process birth rate parameters. The priors set for the remaining parameters were informative and constant for all the runs. Markov-Chain Monte Carlo chains were set to run for 50 million generations, sampling every 2000^th^ generation. Additionally, the MLE chain was set to run for 5 million generations with 200 path steps. Each postulated species tree analysis was run twice to check whether the runs converged and the ESS values were <200. Bayes Factors (2*ln*Bf) were estimated from the MLE (combined from the two runs) to compare species group scenarios and select the most likely scenario [46]: 2*ln* Bf = 0–2 “not worth more than a bare mention”; 2*ln* Bf = 2–6 “positive” support; 2*ln* Bf = 6–10 “strong” support; and 2*ln* Bf *>*10 “decisive” support in distinguishing between competing hypotheses. The phylogenetic trees resulting from the *BEAST runs *per se* are of no importance to BFD, which is why we do not show them here.

### Cytogenetics

Three males of *T*. *curupi* n. sp. from the type locality and nine males of *T*. *uruguayensis* from El Palmar National Park were cytogenetically analyzed. Specimens were carried alive to the laboratory and the testes were dissected in insect saline solution (154 mM NaCl, 5.63 mM KCl, 2.25 mM CaCl_2_, 2.38 mM NaHCO_3_) and incubated in hypotonic solution (75 mM KCl) for 30 min. Tissue fixation, cytogenetic preparations and Giemsa staining were made according to [29]. The C-banding was performed according to the protocol described by [47] and then stained with DAPI (4',6-diamidino-2-phenylindole). Fluorescence in situ hybridization (FISH) was performed as described by [48] for indirect labeling and antibody detection, using 28S rDNA and (TTAGG)_n_ telomeric probes. Both probes were obtained as described by [49] and labelled by PCR with biotin-16-dUTP (Roche). The probes were detected with streptavidin-Cy3 conjugate (Sigma). The preparations were counterstained with DAPI and mounted in Vectashield (Vector). The slides were examined under a Leica DMLB microscope equipped with a Leica DFC350 FX monochrome digital camera. Pictures were pseudo-coloured and processed with Adobe Photoshop CS5. Chromosome measurements were performed on well-spread postpachytene cells using ImageJ software (http://imagej.nih.gov/ij/). The relative length of each chromosome was calculated as a percentage of total diploid complement length (%TCL).

## Results

### Taxonomy

The systematic position of *T*. *curupi* n. sp. as different species is clearly supported by morphological, molecular and cytogenetic data (see below). *Tityus curupi* n. sp. is most closely related to *T*. *uruguayensis*.

**Fig 3:**
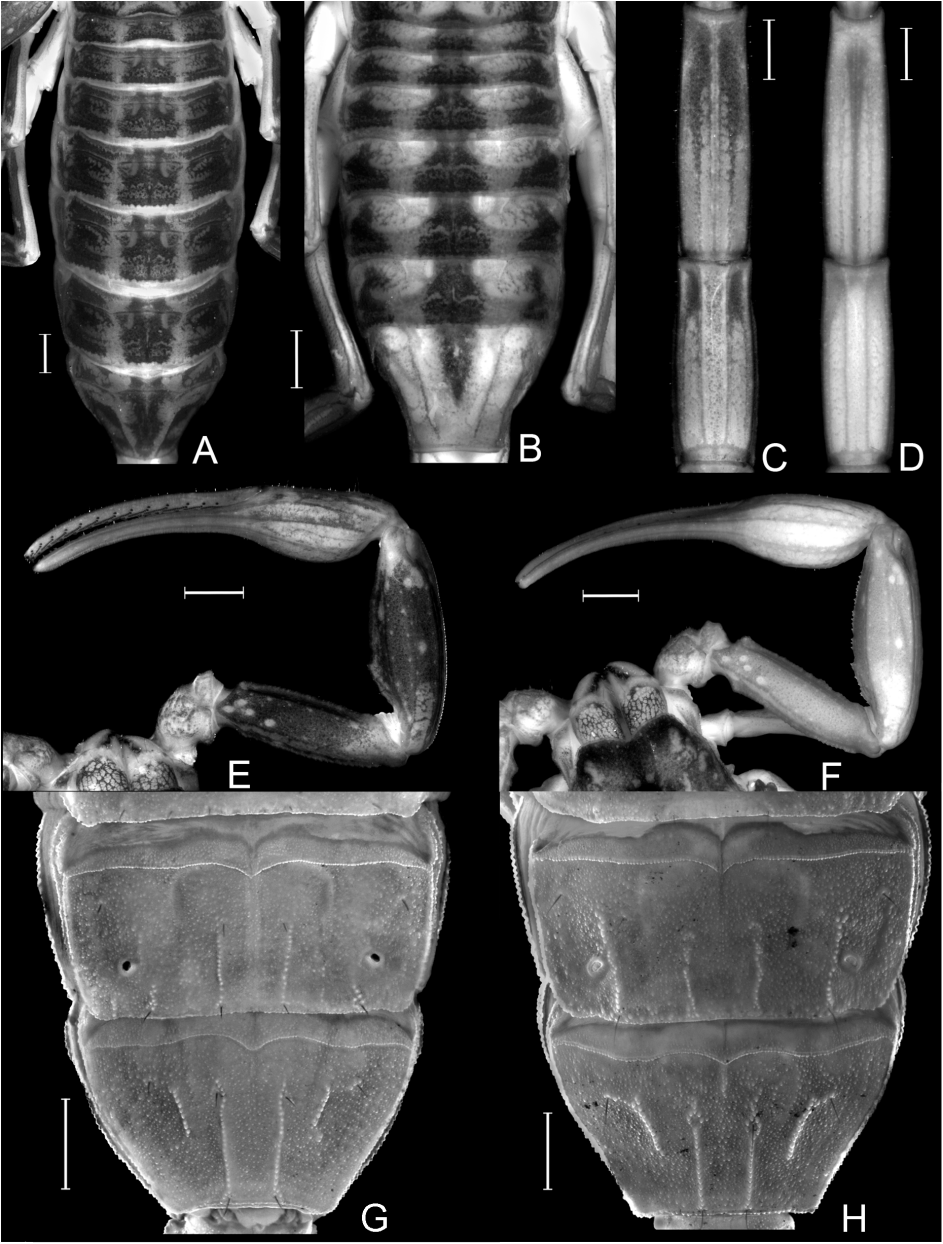
Diagnostic characters. A, C, E, G. Diagnostic characters of *Tityus uruguayensis*. A. Pigment pattern of tergites; C. Pigment pattern of ventral surface of metasoma; E. Pigment pattern of pedipalps; G. Sternites IV and V. B, D, F, H. Diagnostic characters of *Tityus curupi* n. sp. B. Pigment pattern of tergites; D. Pigment pattern of ventral surface of metasoma; F. Pigment pattern of pedipalps; H. Sternites IV and V. Scale bars = 1mm.

**Fig 4:**
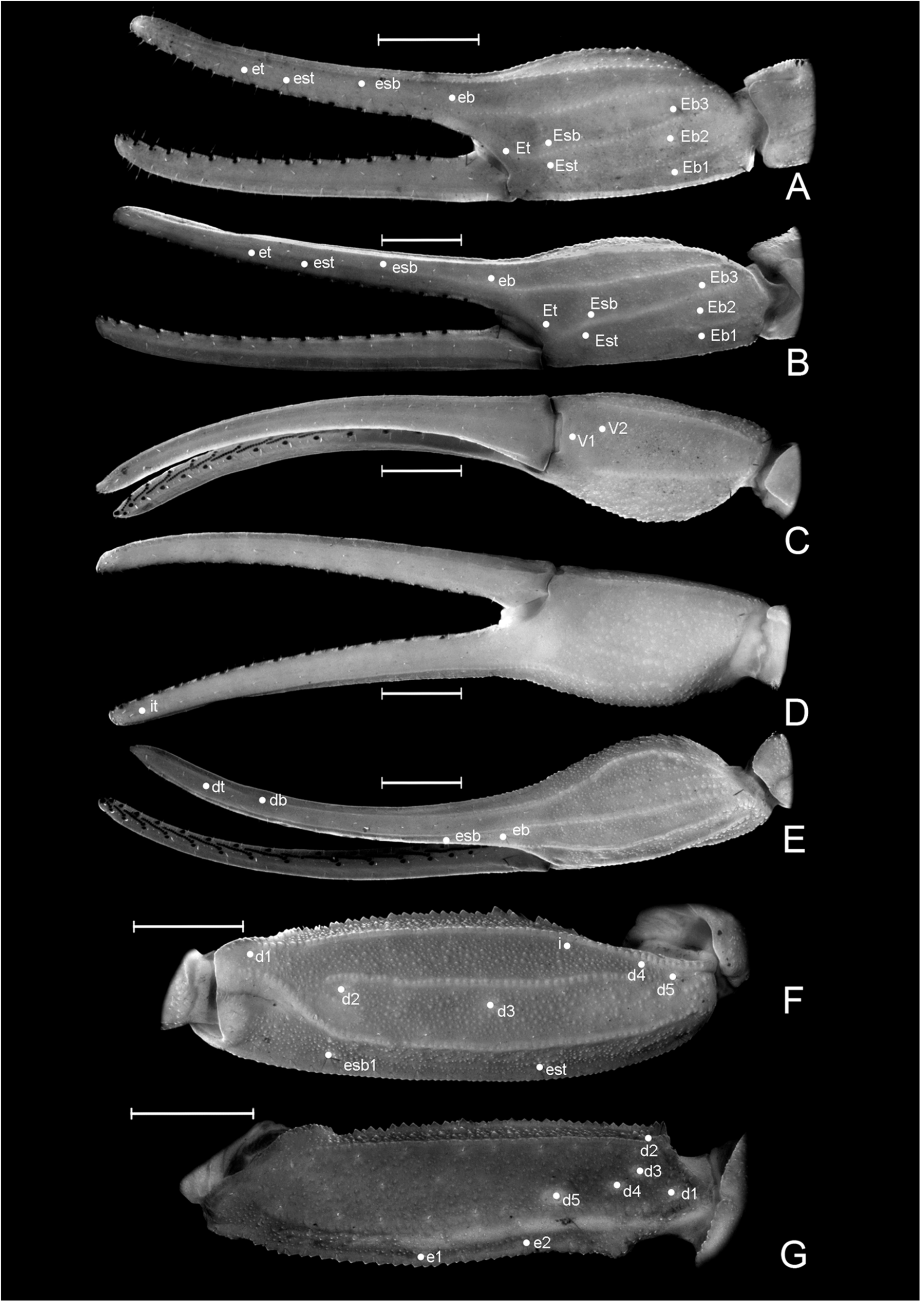
Morphology of *Tityus curupi* n. sp. A−E. Chela. A. Male, external aspect; B. Female, external aspect; C. Female, ventral aspect; D. female, internal aspect; E. Female, ventral aspect. F. Patella, female, dorsal aspect. G. Femur, female, dorsal aspect. Scale bars = 1mm.

**Fig 5:**
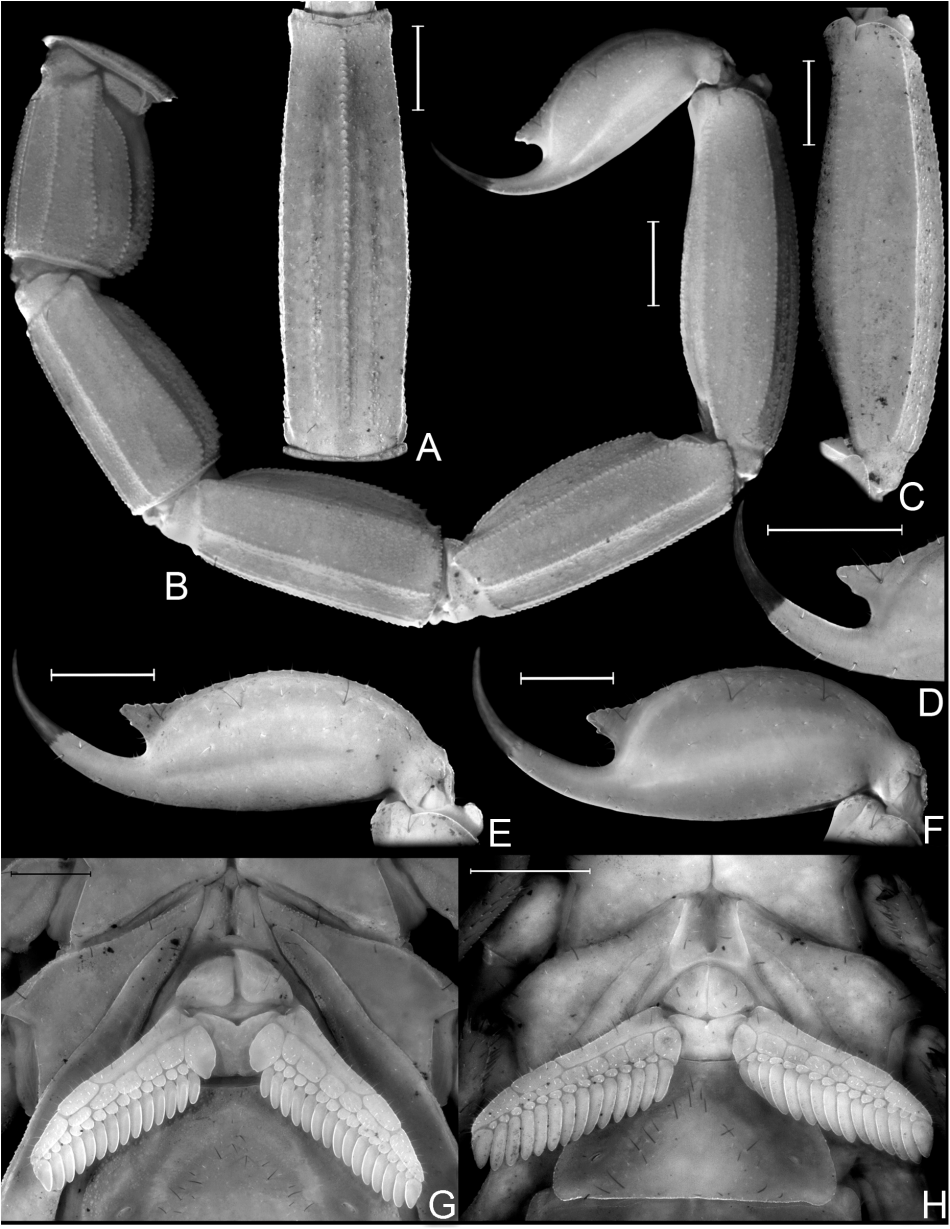
Morphology and Diagnostic characters. A, B, C, E, F, G, H. Morphology of *Tityus curupi* n. sp. A. Metasomal segment V, female, ventral aspect; B. Metasoma, female, lateral aspect; C. Metasomal segment V, male, lateral aspect; E. Telson, female, lateral aspect; F. Telson, male, lateral aspect; G. Pectines, female, ventral aspect; H. Pectines, male, ventral aspect. D. Diagnostic characters of *Tityus uruguayensis*. D. Posterior margin of telson, female, lateral aspect. Scale bars = 1mm.

**Fig 6:**
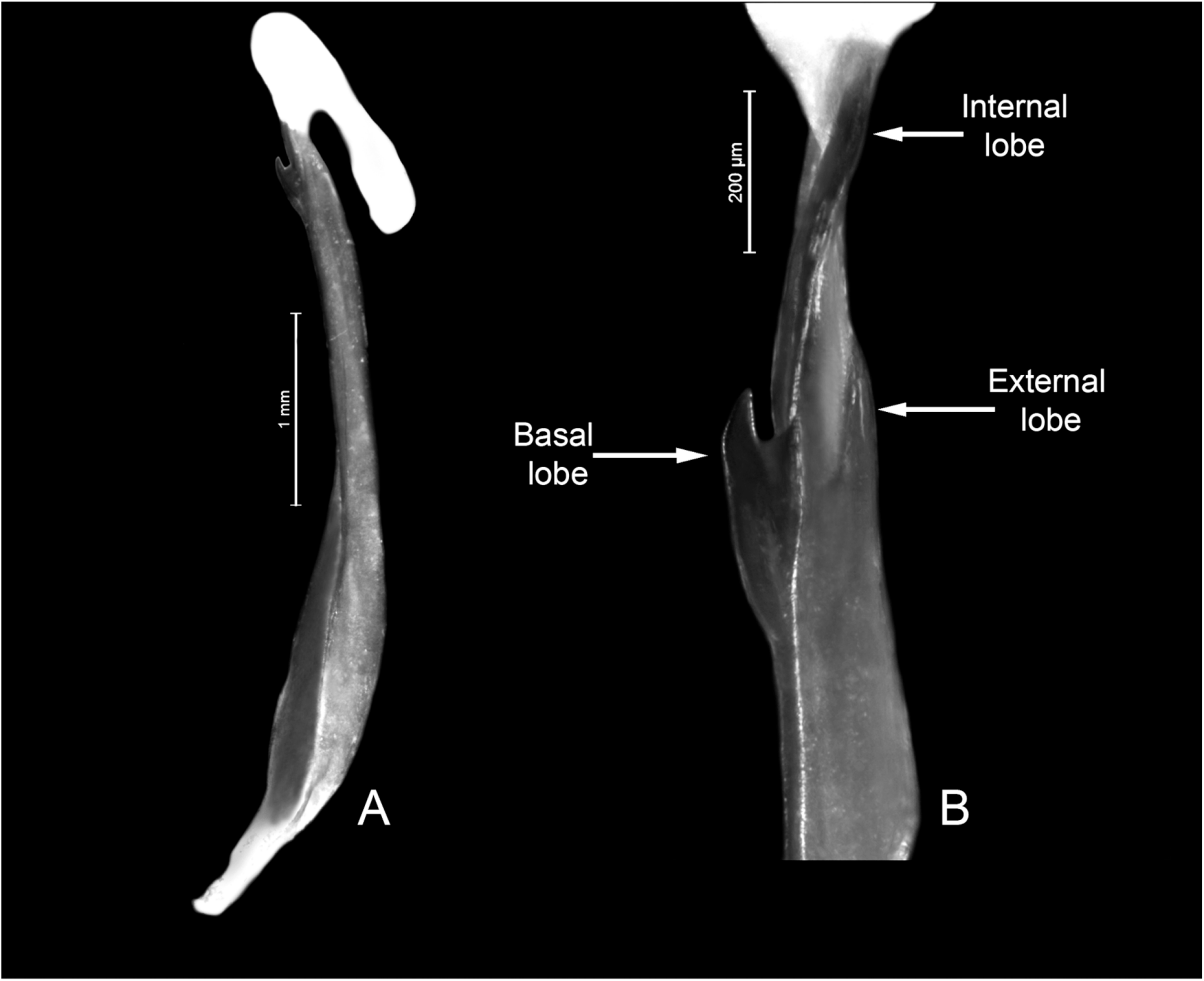
Morphology of *Tityus curupi* n. sp. Right hemispermatophore. A. Ventral aspect; B. Detail of lobe region.

**Fig 7:**
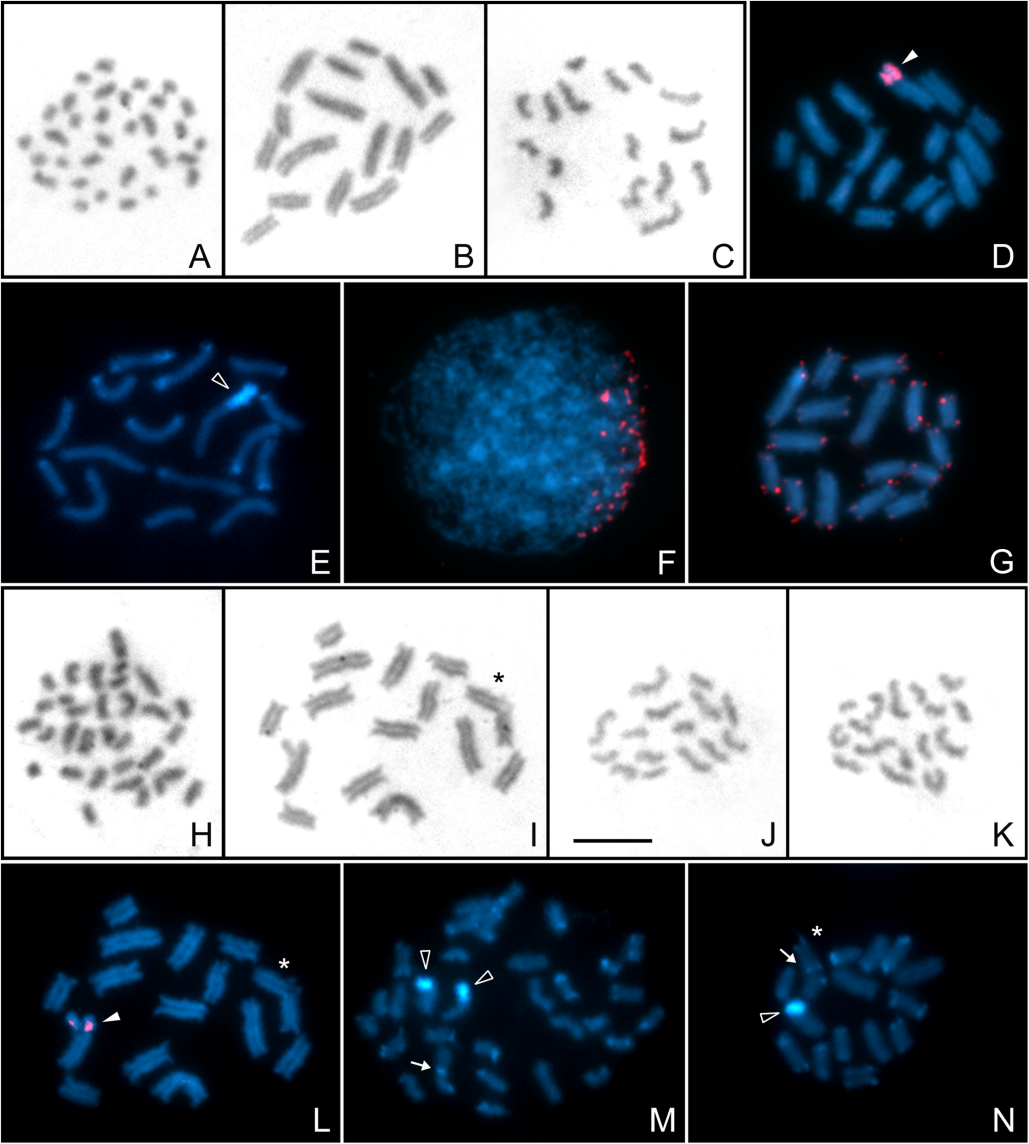
**Male mitosis and meiosis of *Tityus curupi* n. sp. after Giemsa staining (A-C, H-K), C-Banding stained with DAPI (E, M, N), FISH with 28S rDNA (D, L) and (TTAGG)**_***n***_ **telomeric probes (F, G).** A. Mitotic metaphase (2n = 32); B. Prometaphase I (16II); C. Metaphase II (n = 16); D. Prometaphase I; E. Late postpachytene; F. Leptotene/early zygotene with a bouquet configuration of telomeres; G. Prometaphase I; H. Mitotic metaphase (2n = 31); I. Prometaphase I (14II + III); J, K. Metaphase II (n = 15 and n = 16, respectively). L. Prometaphase I (same cell shown in I); M. mitotic late prophase; N. Prometaphase I. White arrowheads indicate 28S rDNA hybridization signals. Empty arrowheads indicate C-bands corresponding to rDNA sites. White arrows indicate interstitial C-band. Asterisks indicate the trivalent. Scale bar = 10 μm.

*Tityus curupi* n. sp. (Figs 1–7; Table 1, A in S1 Table, A in S2 Table; S1 File)

**Table 1:** Measurements (mm) of *Tityus curupi* n. sp. (holotype and paratype, MACN).

	*Tityus curupi* n. sp.	
Specimen		
Type	**Holotype**	**Paratype**
Sex	**♂**	**♀**
Collection	**MACN**	**MACN**
**Carapace:**		
length	3.80	4.87
anterior width	1.93	2.53
posterior width	3.80	5.00
**Chela:**		
length	7.18	8.51
width	1.43	1.27
height	1.53	1.73
**Patella:**		
length	4.33	5.00
width	1.40	1.73
**Femur:**		
length	3.80	4.67
width	1.13	1.33
**Mesosoma:**		
length	10.64	12.37
**Metasomal segment I:**		
length	2.53	3.00
width	2.00	2.40
height	1.73	2.20
**Metasomal segment II:**		
length	3.33	3.40
width	1.73	2.07
height	1.67	2.07
**Metasomal segment III:**		
length	3.67	4.20
width	1.67	2.07
height	1.60	2.00
**Metasomal segment IV:**		
length	4.00	4.80
width	1.60	2.00
height	1.53	1.87
**Metasomal segment V:**		
length	4.67	5.73
width	1.47	1.80
height	1.60	1.87
**Metasoma:**		
total length	22.33	22.56
**Telson:**		
total length	4.13	4.80
vesicle width	1.27	1.60
vesicle height	1.40	1.67
**Total length:**	36.77	39.80

**Type material**: (MACN). Argentina, Corrientes Province, San Martín Department, Paraje Tres Cerros. Holotype ♂: Estancia Las Marías, Cerro Capará (29º09’14.28”S; 56º51’50.14”W), 26/VIII/2014, Cajade coll., (MACN-Ar 35687). *Paratypes*: 1 ♀ (MACN-Ar 35688), 1 juvenile (MACN-Ar 35689), Cerro Chico (29º06’48.85”S; 56º55’05.81”W), 26/VIII/2014, Cajade coll.; 1 juvenile (MACN-Ar 35690), 28/IX/2012, Cajade coll.; 2 juveniles (MACN-Ar 35691 & 35692), Estancia Higuera-Cue, Cerro Chico, 26/VIII/2014; 4 juveniles (MACN-Ar 35693, 35694, 35695, 35696), Estancia Higuera-Cue, Cerro Nazareno (29º06’34.59”S; 56º55’55.27”W), Ojanguren-Affilastro, Adilardi, Piacentini & Ramírez coll., 22-24/II/2015; 1 ♂ (MACN-Ar 35697), 5 ♀ (MACN-Ar 35698, 35699, 35720, 35721, 35722), 3 juveniles (MACN-Ar 35723, 35724, 35725), Estancia Higuera-Cue, Cerro Chico, Ojanguren-Affilastro, Adilardi, Piacentini & Ramírez coll., 22-24/II/2015.

#### Etymology

The name of this species is a noun in apposition referring to the “Curupí”, a goblin of the Guaraní mythology whose legend says that it chases women in the forests when they venture alone to get firewood.

#### Diagnosis and comparisons

Medium sized species of *Tityus* belonging to the *bolivianus* complex. *Tityus curupi* is most closely related to *T*. *uruguayensis*; both species can be separated because the external VSM carinae of tergite V of *T*. *uruguayensis* are short and straight, occupying the median third of the segment (Fig 3G), whereas in *T*. *curupi* these carinae are more developed occupying the median half of the segment, and are curved towards the external margin, forming a semi-arc and partially surrounding the lateral macrosetae (Fig 3H).

The external VSM carinae of tergite IV are also more developed in *T*. *curupi* than in *T*. *uruguayensis*; in *T*. *uruguayensis* these carinae are restricted to the posterior margin of the segment occupying less than a posterior quarter of it (Fig 3G), whereas in *T*. *curupi* they occupy the posterior half or even two thirds, of the segment (Fig 3H).

The subaculear tuberlcle of the telson is also useful to separate *T*. *curupi* n. sp. from *T*. *uruguayensis*; in *T*. *curupi* n. sp. the posterior apex is pointy, the ventral margin clearly serrated and the dorsal paired spines most usually clearly separated (Fig 5E and 5F). In *T*. *uruguayenis*, on the other hand, the distal margin of the subaculear tubercle is dull, the ventral margin, only slightly undulated, and the dorsal paired spines reduced, and most usually fused in a single tiny granule (Fig 5D) [1].

*Tityus curupi* is larger in average size than *T*. *uruguayensis*, total length of males of *T*. *uruguayensis* varies from 25 to 30 mm [1], whereas in males of *T*. *curupi* it varies from 36 to 42 mm; total length of females of *T*. *uruguayensis* varies from 28 to 39 mm [1], whereas in females of *T*. *curupi* it varies from 36 to 47 mm.

The most conspicuous diagnostic characters to separate both species are from the pigment pattern; *T*. *curupi* bears three medium sized spots on the tergites which occupy less than a quarter of the segment each, leaving two longitudinal broad depigmented dorsal stripes (Fig 3B) whereas *T*. *uruguayensis* presents three large dark spots that occupy almost a third of the segment each, leaving only two thin depigmented dorsal stripes (Fig 3A). The dorsal surface of pedipalpal femur and patella of *T*. *uruguayensis* are densely pigmented (Fig 3E), whereas in *T*. *curupi* they are depigmented (Fig 3F). *Tityus uruguayensis* presents two VSM dark spots in the posterior third of metasomal segments, that in metasomal segment V can extend over the VSM carinae reaching the anterior third of the segment (Fig 3C), whereas in *T*. *curupi* the ventral surface of metasomal segments is usually depigmented, or presents abarely visible brownish spot in the posterior margin of each segment, which can extend over the VL carinae on segments I-IV, and over the VM carina in segment V (Fig 3D).

Due to its size and pigment pattern *T*. *curupi* n. sp. could be confused with *Tityus trivittatus* Kraepelin 1898 which occurs in neighbouring areas of lowlands of Corrientes province [1], but not in the hills of Paraje Tres Cerros. Both species can be easily separated because in females of *T*. *trivittatus* the first (or basal) median lamella of pectines is not lobular and is similar in size in females and males [1], whereas in females of *T*. *curupi*. n. sp it is clearly lobular (Fig 5G) and much larger than in males (Fig 5H). Both species can also be separated because the hemispermatophore of *T*. *curupi* n. sp. lacks the median lobe and its external lobe is dorsally fused to the trunk by a thin lamina (Fig 6B), whereas the hemispermatophore of *T*. *trivittatus* bears a median lobe, and its external lobe is not fused dorsally to the trunk of the hemispermatophore [1].

**Description**: Based on the holotype ♂, and paratypes (MACN).

*Total length*: 36.77–42.00 mm (*n* = 2; mean = 39.39) in ♂; 36.00–47.50mm (*n* = 6; mean = 41.70) in ♀. *Color*: Base color yellowish, with dark brown pattern in chelicerae, carapace and tergites (Fig 2B and 2C). Chelicerae: light yellow with a dense reticulate pattern in manus. Carapace: with black areas around ocular tubercle and lateral eyes; with a dark triangle raging from the ocular tubercle to the frontal margin; lateral areas with dark reticular pattern; with two posterolateral dark spots. Tergites I–VI (Fig 3B) each with three triangular dark spots extending the entire length of the segment, two lateral, and a median dark spot; these spots are not connected to each other, leaving two wide unpigmented stripes, however in the posterior margin of the segment between lateral and median spots, there is an area with an orange pigment, different from the yellowish base color. Tergite VII with a lateral faint dark spot on each side, and a median triangular spot extending the anterior two thirds of the segment. Sternites, sternum, genital opercula and pectines unpigmented. Metasomal segments I-IV unpigmented or with a faint brownish spot in the posterior margin of the segment, extending over the VL carinae; segment V unpigmented or with faint brownish pigment in the posterior third of the segment, extending as a ventro-median stripe over the VSM carina (Fig 3D). Telson, vesicle unpigmented, or with faint reddish pattern on ventral surface, acculeus dark reddish-brown. Pedipalps: completely unpigmented in most specimens, but in some specimens some areas with a faint brownish pattern in femur, patella and over pedipalp carinae and fingers (Fig 3F). Legs: unpigmented or with a faint brownish pattern in the distal half of femur and in patella. *Carapace*: Anterior margin with a conspicuous broad median notch that divides the anterior margin into two lobes. Surface densely granular. Anteromedian longitudinal sulcus, interocular sulcus; posteromedian longitudinal and lateral sulci present and conspicuous. Median ocular tubercle well developed, and clearly protruding above carapace in lateral profile; median ocelli well developed, approximately one diameter apart. Three small lateral ocelli on each side of carapace. Anterior median carinae, median ocular carinae, lateral carinae, and posterior carinae all granular and well developed. *Chelicerae*: Tegument granular, especially near the distal margin. Dentition typical for the genus. Teeth well developed. *Pedipalps*: Femur (Fig 4G), surface sparsely granular; DE, DI, VE and VI carinae granular and well developed, extending the entire length of the segment; internal margin with an interno-median (IM) carina extending the entire length of the segment. Patella (Fig 4F) intercarinal surfaces sparsely granular DI, DM, DE, IM VI, VE and EM carinae granular, extending the entire length of the segment. Chela manus slender (s4A−E), DI, DE, DS, D, SD, E, VE, VI, and IM carinae granular and well developed (Fig 4A); fixed and movable fingers thin, elongated and slightly curved internally; movable finger with 14, or more usually 15, sub-parallel denticle rows, being the basal row longitudinal and sometimes formed by the fusion of two denticle rows; fixed finger with 14 or 15 sub-parallel denticle rows, being the basal row longitudinal and always formed by the fusion of two or three denticles rows; each of these denticles rows presents and apical enlarged tooth and two basal enlarged teeth, which are two or three times bigger than the rest of the denticles; the apical enlarged tooth presents a hyaline and a chitinized setae in the base of it; the most apical of the basal enlarged denticles presents a single hyaline setae in its base, and the most basal presents a chitinized setae in its base; at the apical margin of each finger there is an apical tooth with four lamellar setae surrounding its basal margin. Movable finger with a basal lobe poorly developed, slightly more so in ♂; fixed finger with a small shallow notch facing the basal lobe. Trichobothrial pattern Orthobothriotaxic Type A; femur with 11 trichobothria, α configuration and trichobothria *D*_*2*_ placed on the internal surface (Fig 4G); patella with 13 trichobothria (Fig 4F); chela with 15 trichobothria, being *esb* petit (Fig 4A–4E). *Legs*: Intercarinal surfaces granular. Carinae granular, extending the entire length of the segment. Basitarsi each with two well-developed, equal-length pedal spurs; the external one presents an external, very well developed, macroseta. Telotarsi short, ventrally with abundant chitinized setae, and some VL hyaline setae with following counts, on legs I and II: 4/3, III and IV: 4/4.Ungues well developed, equal in length and curved. *Sternum*: Sub-triangular (Fig 5G and 5H). *Genital opercula*: Medium sized. Sclerites subtriangular, in ♀ the posterior margin of the sclerites is perpendicular to the axis of the body, whereas in ♂ it forms an acute angle (Fig 5G and 5H). *Pectines*: With a single row of median lamellae; first median lamella conspicuously more enlarged in females than in males (Fig 5G and 5H). Fulcra present, small and subcircular. Pectinal teeth medium sized; tooth count: 14–15 in ♂ (*n* = 6; median = 15), 13–16 in ♀ (*n* = 7; median = 15). *Tergites*: Pretegites with fine granulation, separated from the rest of the tergite by a well-developed transversal carina. Tergites I–VI, surfaces granular becoming more coarsely so in the posterior half of the segment; with three longitudinal carinae, one Dorsal-Median extending the entire length of the segment and two Dorso-submedian in the anterior third of the segment, joining the transversal carina of pretergite, and forming a semi-arc. The dorsal median carina is formed by a single row of granules that becomes double in its median part. Tergite VII surfaces granular, Dorsal-median carina extending the anterior two thirds of the segment, formed by a double row of granules in its anterior half, fusing in a single row in the second half; with four longitudinal Dorso-submedian carinae extending the entire length of the segment, fusing anteriorly to each other in a subcircular transversal carinae, that also fuses in its anterior margin with the transversal carina of pretergite. *Sternites*: Sternite I finely granular near the lateral margins, with two VSM furrows joining in the anterior margin and surrounding a glandular subtriangular area, also delimitated by a row of setae, this gland is more conspicuous in males than in females; with small, elliptical spiracles. Sternite II: tegument finely granular, with scattered setae and two small VSM furrows reduced to an indent, with small, elliptical spiracles. Sternite III: similar to II but with a posteromedian, subtriangular gland, more conspicuous in males. Sternite IV: similar to II but with four longitudinal carinae, formed by a single row of granules, the external carinae occupy the posterior two thirds of the segment, whereas the internal carinae occupy the posterior half of the segment. Sternite V: tegument granular, with four VSM carinae (Fig 3H), the external carinae are placed in the median part of the segment and forming a semi-arc towards the external margin, the internal carinae are longitudinal and placed in the posterior two thirds of the segment; in some specimens there is a poorly developed VM carina in the anterior third of the segment. *Metasoma*: Metasomal segment I: intercarinal surfaces finely granular; DL, LSM, LIM, VL and VSM carinae granular end extending the entire length of the segment; distal granules of the DL carinae slightly spinoid (Fig 5B); between DL and LSM carinae there is an intermediate poorly developed carina in some specimens. Segment II similar to segment I but LIM carina is only present in the posterior half of the segment. Segments II and IV similar to segment II but without LIM carina. Segment V, elongated (Fig 5C), dorsolateral margin with a poorly developed LSM carina, VL and VM carinae granular and extending the entire length of the segment (Fig 5A); with poorly developed VSM carinae, formed by tiny granules, but extending almost the entire length of the segment. *Telson*: Vesicle oval, being slightly more globose in females (Fig 5E and 5F). Surface smooth, except for a poorly developed VM carina and some scattered setae. Subaculear tubercle subtriangular, inferior margin partially serrated and connecting to the VM carina, base of the superior margin with two granules that in some specimens are fused to each other. Aculeus strongly curved. *Hemispermatophore*: Slender (Fig 6A). Pars reflexa poorly developed. Lobe region poorly developed, Internal lobe short, Basal lobe hook-like, External lobe barely visible, connected to the trunk of the hemispermatophore by a thin cuticular lamina (as in the rest of the species of the *bolivianus* complex) (Fig 6B).

#### Distribution and ecology

*Tityus curupi* n. sp. has only been collected in its type locality in the low hill range of Paraje Tres Cerros, in north eastern Corrientes province, in northern Argentina (Fig 2). *Tityus curupi* n. sp. has been collected in the three hills of the area (Figs 1A and 2B–2D), but not in the surrounding grasslands and forests. *Tityus curupi* n. sp. is a litophilous species which only occurs in the rocky reliefs of the hills. At night most specimens were observed hunting in crevices between stones. *Tityus curupi* n. sp. is in sympatry with only one scorpion species *Bothriurus jesuita* Ojanguren-Affilastro 2003; however this bothriurid, which is common in northeastern Argentina [50, 51], occupies a different microhabitat, being more common in the plains surrounding the hills.

### Species delimitation

The genetic distances between *T*. *curupi* n. sp. and *T*. *uruguayensis* given as net evolutionary divergences were of 0.096 for COI, 0.119 for 16S and 0.002 for 28S. Based on the Bayes Factor species Delimitation (BFD) analyses with path sampling (PS) and stepping-stone sampling (SS), *T*. *curupi* n. sp. and *T*. *uruguayensis* are two different species. The Marginal Likelihood Estimate (MLE) using PS was -6683.2 when considering the two as conspecific and -6659.8 when separating them. Similarly, using SS the MLE values were -6677.82 and -6659.24 for one and two species, respectively. Hence in both cases, the 2-fold difference between the MLE values is greater than 10, indicating decisive support for the 2-species hypothesis.

### Cytogenetics

#### *Tityus curupi* n. sp.

Two males of *T*. *curupi* n. sp. presented 2n = 32 holokinetic chromosomes at mitosis (Fig 7A). Postpachytene cells showed 16 achiasmatic bivalents (16II) (Fig 7B), compounded of chromosomes decreasing in size from 4.4 to 2.2% of the TCL (Table A in S2 Table). Cells at second meiotic division presented n = 16 chromosomes (Fig 7C). FISH with 28S rDNA probes revealed conspicuous signals at the terminal region of both chromosomes of the second largest bivalent, comprising almost one third of its length (Fig 7D). C-banding revealed conspicuous bands at the terminal region of one bivalent, that colocalize with the rDNA site and small bands at the terminal regions of almost all the other chromosomes (Fig 7E). FISH with (TTAGG)_*n*_ telomeric probes revealed a *bouquet* configuration at leptotene/early zygotene (Fig 7F). At postpachytene, telomeric signals were detected at the terminal regions of all chromosomes, without any interstitial signals (Fig 7G). The other male presented 2n = 31 chromosomes (Fig 7H), and at meiosis I showed 14 bivalents and one trivalent (14II+III); the latter consist of a large chromosome (similar in size to the chromosomes of the larger bivalents) and two smaller chromosomes (Fig 7I). Cells at second meiotic division presented n = 15 or n = 16 chromosomes (Fig 7J and 7K). This cytotype showed rDNA sites with the same features of the cytotype with only bivalents (Fig 7L). C-banding pattern was similar to that observed in the cytotype with only bivalents, plus one interstitial C-band in a large chromosome (Fig 7M), that corresponds to the large chromosome of the trivalent (Fig 7N).

#### Tityus uruguayensis

**Fig 8:**
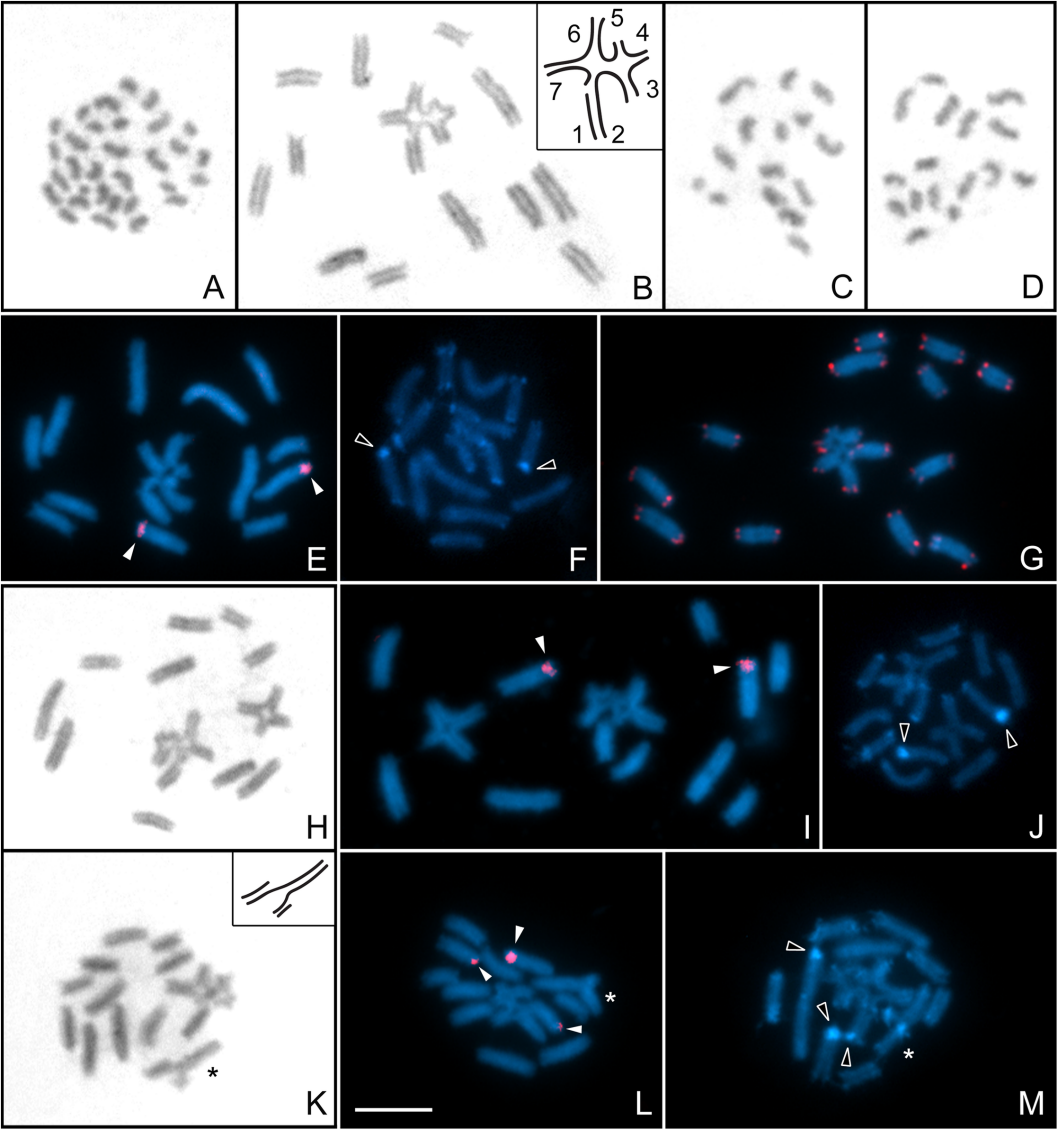
**Male mitosis and meiosis of *Tityus uruguayensis*. after Giemsa staining (A-D, H, K), C-Banding stained with DAPI (F, J, M), FISH with 28S rDNA (E, I, L) and (TTAGG)**_***n***_ **telomeric probes (G).** A. Mitotic metaphase (2n = 31); B. Prometaphase I (12II+VII). Inset: scheme of the heptavalent; C, D. Metaphase II cells of a male with 12II+VII (n = 15 and n = 16, respectively); E, F, G. Prometaphase I (12II+VII); H, I, J. Prometaphase I (10II+RIV+VII); K. Prometaphase I (10II+CIV+VII). Inset: scheme of the chain quadrivalent; L, M. Prometaphase I. White arrowheads indicate 28S rDNA hybridization signals. Empty arrowheads indicate C-bands corresponding to rDNA sites. Asterisks indicate the chain quadrivalent. Scale bar = 10 μm.

The nine males of *T*. *uruguayensis* presented 2n = 31 holokinetic chromosomes at mitosis, similar in size to the ones in *T*. *curupi* n. sp. (Fig 8A). All the males presented a heptavalent at postpachytene, compounded of chromosomes of uneven size: two large of different size (5.3 and 4.1% of the TCL), four medium (3.0, 3.1, 2.5 and 2.3% of the TCL) and one small chromosome (1.7% of the TCL), which were numbered one to seven (see inset in Fig 8B; Table B in S2 Table). In seven of these males the remaining chromosomes appeared as bivalents (12II+VII) (Fig 8B), compounded of chromosomes decreasing in size from 4.3 to 2.3% of the TCL (Table B in S2 Table). Each of the other two males showed 10 bivalents plus different quadrivalents: one male presented a ring quadrivalent formed by chromosomes of similar size (10II+RIV+VII) (Fig 8H), while the other presented a chain quadrivalent composed of two large, one medium and one small chromosome (10II+CIV+VII) (Fig 8K). Cells at second meiotic division presented n = 15 or n = 16 chromosomes in all the analyzed males (Fig 8C and 8D). FISH with 28S rDNA probes revealed signals at the terminal region of two chromosome pairs that constitute two bivalents at meiosis I in all cytotypes (Fig 8E, 8I and 8L). In the chain quadrivalent cytotype, an extra signal was detected in one terminal region of the second chromosome of the heptavalent (Fig 8L). C-banding revealed conspicuous terminal bands that colocalized with the ribosomal clusters and smaller bands at the terminal region of almost all chromosomes in the three cytotypes (Fig 8F, 8J and 8M). FISH with (TTAGG)_*n*_ telomeric probes also revealed a *bouquet* configuration at leptotene/early zygotene and at postpachytene, telomeric signals were exclusively detected at the terminal regions of all chromosomes (Fig 8G).

## Discussion

*Tityus curupi* n sp. is closely related to *T*. *uruguayensis* as well as to the rest of the species of the *bolivianus* complex, and is the second known species of this group not occurring in the Andes. The particular disjunct distribution of this species complex may be related with the mid-Miocene marine ingressions that took place in the intermediate area where it is currently absent. This complex of species most likely presented an ancient continuous distribution, ranging from central and northern Andes, to the Atlantic coast of Uruguay. The marine ingressions apparently isolated the species of this complex (Andean and lowlands) that remained on both sides of this ancient sea. The low hills of Paraje Tres Cerros, where *T*. *curupi* occurs, apparently remained alongside these marine floods. Due to this, and to its particular geomorphology, clearly different from the surrounding areas, several endemic species of animals and plants have evolved there [18, 19, 20, 13, 14]. Our group recently published a dated molecular phylogeny of South American Buthidae which strongly supports this hypothesis [11]. In that contribution we also propose a recent and relatively fast (5 MY) colonization pattern of the intermediate emerged areas of Chaco, by several groups of scorpions of northern lineages. These are buthids of genus *Zabius*, and *Tityus* belonging to *trivittatus* and *clathratus* groups. This colonization pattern of Chaco is also temporarily coincident with that we have also obtained for Chacoan bothriurid Scorpions of sub-genus *Ministernus* of genus *Brachistosternus* [52], providing more support to our hypothesis.

*Tityus curupi* n. sp. (2n = 32, 31) and *T*. *uruguayensis* (2n = 31) are the two species of *Tityus* with the highest chromosome numbers known at present. These diploid numbers are exceptionally high, which would also cytogenetically support the close relationship between these two species. The other studied species of genus *Tityus* show diploid complements ranging from 5 to 20 chromosomes, except *Tityus neglectus* Mello-Leitão 1932, with 2n = 27 [21, 22, 23, 24, 25, 26, 27, 28, 29]. These species show different configurations at meiosis I. The specimens of *T*. *curupi* n. sp. with 2n = 32 present only bivalents, while the specimen with 2n = 31 shows bivalents and one trivalent. The variation of chromosome numbers in this species could have originated by the fusion of two chromosomes of two of the smaller pairs, giving rise to a large chromosome in the specimen with 2n = 31. This hypothesis is also supported by the presence of an interstitial block of constitutive heterochromatin in this large chromosome that could represent the relict of terminal heterochromatin of the fused chromosomes. *Tityus uruguayensis* presents different multivalents (VII and two types of IV), not associated with variation in chromosome number. In this species the fixed heptavalent could have originated by one fusion (that reduced the diploid number to an odd one) and at least two reciprocal translocations. The origin of the ring quadrivalent present in one male can be explained by one reciprocal translocation, while the origin of the chain quadrivalent in the other male is certainly more complex. It could be postulated that the first step was the unequal fragmentation of a large pair finally giving rise to two bivalents of different size (medium and small) and a diploid number of 33. Later, two different fusions involving these two pairs and another large pair took place: one large chromosome fused with one medium chromosome, while the other large homologue fused with one small chromosome, thus reducing the diploid number again to 31.

Buthidae species have, as a rule, one terminal rDNA locus in only one pair of chromosomes, associated with a large heterochromatic block [28, 29, 49, 53, 54]. This is a remarkable feature, given the high incidence of intra- and interspecific structural rearrangements and variation in chromosome number. This rDNA pattern was also found in *T*. *curupi* n. sp. herein analyzed.

Variations in rDNA localization and number are unusual in family Buthidae. Four species of genus *Androctonus* Ehrenberg 1828 show one pair of interstitial rDNA sites, associated with a secondary constriction [55], while only *Tityus obscurus* (Gervais 1843) presented terminal rDNA sites in two chromosome pairs [56].

The case of *T*. *uruguayensis* is remarkable, since it is the second analyzed species of Buthidae that presents rDNA sites in two chromosome pairs, besides the presence in one specimen of an additional rDNA site in one chromosome of the heptavalent. Additional rDNA sites can be the result of different mechanisms as chromosome rearrangements, transposition or ectopic recombination [57], which could be the case of this species, since the presence of two pairs of chromosomes and an extra chromosome with rDNA loci seems to be a derived characteristic. It is noteworthy that none of the two NOR pairs are involved in the structural rearrangements of this population. The number of rDNA sites in *T*. *uruguayensis* could be characteristic of this species, as well as in *T*. *obscurus* [56]. Therefore, the most striking cytogenetic difference between *T*. *uruguayensis* and *T*. *curupi* n. sp. is the number of NOR pairs that characterize each species.

The specimens of *T*. *curupi* n. sp. present the typical meiotic behavior with all bivalents, or the minimal number of possible rearrangements (one fusion), suggesting that the adaptation of this species to the biogeographic island condition of Paraje Tres Cerros did not require large rearrangements in heterozygosity, as was postulated for other buthid species [58]. The probable anthropic origin of the analyzed population of *T*. *uruguayensis* [1] is supported by the fact that all individuals present a fixed complex heptavalent, which suggests that the population could have originated from a reduced number of individuals.

*Tityus curupi* n. sp. and *Tityus uruguayensis* showed an unusually high chromosome number and a reduced chromosome size, considering the other *Tityus* species hitherto analyzed [26, 28, 54]. These characteristics could indicate that in lowlands species of the *bolivianus* complex, chromosome fragmentations could be involved in karyotype evolution. Schneider et al. [59] similarly proposed for a polymorphic population of *Tityus bahiensis* that the increase in the chromosome number is associated with the decrease in chromosome size, and vice versa, as is usual in organisms with holokinetic chromosomes [60].

Taking into account the karyotype similarities in both herein studied species, and the rearrangements proposed for the meiotic variations, it could be postulated that 2n = 32 is the diploid number of the ancestor of the lowlands species of the *bolivianus* complex. Further cytogenetic analyses of populations of *T*. *uruguayensis* from its original area of distribution are necessary to know the cytogenetic variability of the species, as well as to support our previous hypothesis. The high divergence of chromosome numbers between *T*. *argentinus* (2n = 9, 10) [30], the only Andean species of the *bolivianus* group cytogenetically analyzed, and both lowlands species, could be indicative of a process of major chromosome rearrangements since the separation of both groups. Further cytogenetic analyses of the Andean species are needed to reconstruct the karyotype evolution within the *bolivianus* group.

This contribution is the first scorpion description dealing with so many approaches simultaneously. Hopefully, this kind of multidisciplinary contributions can provide more tools to separate closely related species belonging to groups with a problematic taxonomy, as those of genus *Tityus*.

## Supporting information

S1 Tables **Table A.** Accession Numbers for tissue samples from which DNA sequences were used in this study, deposited in the Colección Aracnológica of the Museo Argentino de Ciencias Naturales “Bernardino Rivadavia” (MACN-Ar), and GenBank accession codes for the D3 region of the 28S rDNA (28S-D3), 16S rDNA (16S) and Cytochrome c Oxidase Subunit I (COI) sequences from all individuals used in the species delimitation analysis. **Table B.** Primers used for PCR amplification of gene fragments for the Bayes Factor species delimitation analyses of *Tityus curupi*.
(DOC)

S2 Tables **Table A.** Chromosome measurements of postpachytene cells of *Tityus curupi* n. sp (2n = 32, 16II). Relative lengths expressed as percentage of total diploid complement length (%TCL). Mean values of N = 15 measured cells and their standard deviations (SD) are given. (*) indicate the rDNA bearing bivalent. **Table B.** Chromosome measurements of postpachytene cells of *Tityus uruguayensis* (2n = 31, 12II+VII). Relative lengths expressed as percentage of total diploid complement length (%TCL). Mean values of N = 10 measured cells and their standard deviations (SD) are given. Chromosomes of the heptavalent are numbered as in inset of Fig 7B.
(DOC)

S1 File List of the studied material and Accession Numbers of the Coleccion Aracnológica of the Museo Argentino de Ciencias Naturales “Bernardino Rivadavia” (MACN-Ar) of the newly sequenced specimens used in the species delimitation analysis. (DOC)
