# Supplementary material for: Multiple approaches to understanding the taxonomic status of an enigmatic new scorpion species of the genus Tityus (Buthidae) from the biogeographic island of Paraje Tres Cerros (Argentina)
Source: PLoS One. 2017 Jul 26;12(7):e0181337. doi: 10.1371/journal.pone.0181337 (PMC5529008; doi:10.1371/journal.pone.0181337)
Supplement: S1 Tables — Table A. Accession Numbers for tissue samples from which DNA sequences were used in this study, deposited in the Colección Aracnológica of the Museo Argentino de Ciencias Naturales “Bernardino Rivadavia” (MACN-Ar), and GenBank accession codes for the D3 region of the 28S rDNA (28S-D3), 16S rDNA (16S) and Cytochrome c Oxidase Subunit I (COI) sequences from all individuals used in the species delimitation analysis. Table B. Primers used for PCR amplification of gene fragments for the Bayes Factor species delimitation analyses of Tityus curupi. (DOC) [file pone.0181337.s001.doc]

**S1 Tables**

**Table A.** Accession Numbers for tissue samples from which DNA sequences were used in this study, deposited in the Colección Aracnológica of the Museo Argentino de Ciencias Naturales “Bernardino Rivadavia” (MACN-Ar), and GenBank accession codes for the D3 region of the 28S rDNA (28S-D3), 16S rDNA (16S) and Cytochrome c Oxidase Subunit I (COI) sequences from all individuals used in the species delimitation analysis.

| **Species** | **MACN-Ar** | **28S-D3** | **16S** | **COI** |
| --- | --- | --- | --- | --- |
| *Ananteris balzani* Thorell, 1891 | 35701 | KY674470 | KY674448 | KY674491 |
| *Tityus argentinus* Borelli 1899 | 35705 | KY674474 | KY674452 | KY674493 |
| *Tityus bahiensis* (Perty 1833) | 35706 | KY674475 | KY674453 | ----- |
| *Tityus confluens* Borelli 1899 | 35709 | KY674478 | KY674456 | KY674496 |
| *Tityus uruguayensis* Borelli 1901 | 35714 | KY674433 | KY674425 | KY674442 |
| *Tityus uruguayensis* Borelli 1901 | 35715 | KY674484 | KY674462 | KY674502 |
| *Tityus uruguayensis* Borelli 1901 | 35716 | KY674434 | KY674426 | KY674443 |
| *Tityus uruguayensis* Borelli 1901 | 35717 | KY674435 | KY674427 | KY674444 |
| *Tityus uruguayensis* Borelli 1901 | 35718 | KY674436 | KY674428 | KY674445 |
| *Tityus uruguayensis* Borelli 1901 | 35719 | ----- | ----- | KY674446 |
| *Tityus paraguayensis* Kraepelin 1895 | 35711 | KY674481 | KY674459 | KY674499 |
| *Tityus trivittatus* Kraepelin 1898 | 35713 | KY674483 | KY674461 | KY674501 |
| *Tityus curupi* n. sp. | 35723 | KY674429 | KY674421 | KY674437 |
| *Tityus curupi* n. sp. | 35724 | KY674479 | KY674457 | KY674497 |
| *Tityus curupi* n. sp. | 35693 | KY674430 | KY674422 | KY674438 |
| *Tityus curupi* n. sp. | 35694 | KY674431 | KY674423 | KY674439 |
| *Tityus curupi* n. sp. | 35695 | KY674432 | KY674424 | KY674440 |
| *Tityus curupi* n. sp. | 35696 | ----- | ----- | KY674441 |
| *Zabius birabeni* Mello-Leitão 1938 | 36499 | KY674485 | KY674463 | KY674503 |
| *Zabius fuscus* Thorell 1893 | 35676 | KY674486 | KY674464 | KY674504 |
| *Zabius* sp1 | 36495 | KY674487 | KY674465 | KY674505 |
| *Zabius* sp2 | 36496 | KY674488 | KY674466 | KY674506 |

**Table B.** Primers used for PCR amplification of gene fragments for the Bayes Factor species delimitation analyses of *Tityus curupi*

| **Marker** | **Primer name** | **Primer sequence (5’ to 3’)** | **Reference** |
| --- | --- | --- | --- |
| COI | LCOI 1490 | GGTCAACAAATCATAAAGATATTGG | Folmer *et al.* (1994) |
|  | HCO extern B | CCTATTGAWARAACATARTGAAAATG | Arango & Wheeler (2007) |
| 16S | 16S ar | GTGCAAAGGTAGCATAATCA | Gantenbein *et al*. (1999) |
|  | 16S B | CCGGTTTGAACTCAGATC | Simon *et al.* (1994) |
| 28S | 28S a | GACCCGTCTTGAAGCACG | Borda & Siddall (2004) |
|  | 28S b out | CCCACAGCGCCAGTTCTGCTTACC | Prendini *et al.* (2005) |

**References**

**Arango CP, Wheeler WC. 2007.** Phylogeny of the sea spiders (Arthropoda, Pycnogonida) based on direct optimization of six loci and morphology. *Cladistics,* 23: 255–293.

**Borda E, Siddall ME. 2004.** Arhynchobdellida (Annelida: Oligochaeta: Hirudinida):phylogeneticrelationships and evolution. *Molecular Phylogenetics and Evolution*, 30, 213–225.

**Folmer O, Black M, Hoeh W, Lutz R, Vrijenhoek RC. 1994.** DNA primers for amplification of mitochondrial Cytochrome c Oxidase subunit I from diverse metazoan invertebrates. *Molecular Marine Biology and Biotechnology,* 3: 294–299.

**Gantenbein B, Fet V, Largiadèr CR, Scholl A. 1999.** First DNA phylogeny of *Euscorpius*Thorell, 1876 (Scorpiones, Euscorpiidae) and its bearing on taxonomy and biogeographic of this genus. *Biogeographica,* 75: 49–65.

**Prendini L, Weygoldt P, Wheeler WC. 2004.** Systematics of the Damon variegatus group of African whip spiders (Chelicerata: Amblypygi): Evidence from behaviour, morphology and DNA. *Organisms Diversity & Evolution*, 5, (3): 203–236.

**Simon C, Frati F, Beckenbach A, Crespi B, Liu H, Flook P. 1994.** Evolution, weighting, and phylogenetic utility of mitochondrial gene sequences and a compilation of conserved polymerase chain reaction primers. *Annals of the Entomological Society of America,* 87: 651–701.
