# Supplementary material for: Multiple approaches to understanding the taxonomic status of an enigmatic new scorpion species of the genus Tityus (Buthidae) from the biogeographic island of Paraje Tres Cerros (Argentina)
Source: PLoS One. 2017 Jul 26;12(7):e0181337. doi: 10.1371/journal.pone.0181337 (PMC5529008; doi:10.1371/journal.pone.0181337)
Supplement: S2 Tables — Table A. Chromosome measurements of postpachytene cells of Tityus curupi n. sp (2n = 32, 16II). Relative lengths expressed as percentage of total diploid complement length (%TCL). Mean values of N = 15 measured cells and their standard deviations (SD) are given. (*) indicate the rDNA bearing bivalent. Table B. Chromosome measurements of postpachytene cells of Tityus uruguayensis (2n = 31, 12II+VII). Relative lengths expressed as percentage of total diploid complement length (%TCL). Mean values of N = 10 measured cells and their standard deviations (SD) are given. Chromosomes of the heptavalent are numbered as in inset of Fig 7B. (DOC) [file pone.0181337.s002.doc]

**S2 Tables**

**Table A.** Chromosome measurements of postpachytene cells of *Tityus curupi* n. sp (2n=32, 16II). Relative lengths expressed as percentage of total diploid complement length (%TCL). Mean values of N=15 measured cells and their standard deviations (SD) are given. (*) indicate the rDNA bearing bivalent.

| Chromosome pairs | %TCL | SD |
| --- | --- | --- |
| 1 | 4.4 | 0.2 |
|  | 4.4 | 0.2 |
| 2(*) | 4.1 | 0.2 |
|  | 4.1 | 0.2 |
| 3 | 4.1 | 0.2 |
|  | 4.1 | 0.2 |
| 4 | 3.8 | 0.2 |
|  | 3.8 | 0.2 |
| 5 | 3.5 | 0.1 |
|  | 3.5 | 0.1 |
| 6 | 3.3 | 0.1 |
|  | 3.3 | 0.1 |
| 7 | 3.2 | 0.1 |
|  | 3.2 | 0.1 |
| 8 | 3.0 | 0.1 |
|  | 3.0 | 0.1 |
| 9 | 2.9 | 0.1 |
|  | 2.9 | 0.1 |
| 10 | 2.8 | 0.1 |
|  | 2.8 | 0.1 |
| 11 | 2.7 | 0.1 |
|  | 2.7 | 0.1 |
| 12 | 2.6 | 0.1 |
|  | 2.6 | 0.1 |
| 13 | 2.6 | 0.1 |
|  | 2.6 | 0.1 |
| 14 | 2.5 | 0.1 |
|  | 2.5 | 0.1 |
| 15 | 2.3 | 0.1 |
|  | 2.3 | 0.1 |
| 16 | 2.2 | 0.1 |
|  | 2.2 | 0.1 |

**Table B.** Chromosome measurements of postpachytene cells of *Tityus uruguayensis* (2n=31, 12II+VII). Relative lengths expressed as percentage of total diploid complement length (%TCL). Mean values of N=10 measured cells and their standard deviations (SD) are given. Chromosomes of the heptavalent are numbered as in inset of Fig. 7b.

| Chromosome pairs | %TCL | SD |
| --- | --- | --- |
| 1 | 4.3 | 0.3 |
|  | 4.3 | 0.3 |
| 2 | 4.0 | 0.2 |
|  | 4.0 | 0.2 |
| 3 | 3.7 | 0.1 |
|  | 3.7 | 0.1 |
| 4 | 3.6 | 0.1 |
|  | 3.6 | 0.1 |
| 5 | 3.5 | 0.1 |
|  | 3.5 | 0.1 |
| 6 | 3.3 | 0.1 |
|  | 3.3 | 0.1 |
| 7 | 3.1 | 0.1 |
|  | 3.1 | 0.1 |
| 8 | 3.1 | 0.1 |
|  | 3.1 | 0.1 |
| 9 | 2.9 | 0.1 |
|  | 2.9 | 0.1 |
| 10 | 2.7 | 0.1 |
|  | 2.7 | 0.1 |
| 11 | 2.5 | 0.1 |
|  | 2.5 | 0.1 |
| 12 | 2.3 | 0.3 |
|  | 2.3 | 0.3 |
| Chromosomes of the heptavalent |  |  |
| 1 | 2.3 | 0.2 |
| 2 | 5.3 | 0.4 |
| 3 | 2.5 | 0.3 |
| 4 | 1.7 | 0.2 |
| 5 | 3.1 | 0.2 |
| 6 | 4.1 | 0.3 |
| 7 | 3.0 | 0.4 |
