# Supplementary material for: Multiple approaches to understanding the taxonomic status of an enigmatic new scorpion species of the genus Tityus (Buthidae) from the biogeographic island of Paraje Tres Cerros (Argentina)
Source: PLoS One. 2017 Jul 26;12(7):e0181337. doi: 10.1371/journal.pone.0181337 (PMC5529008; doi:10.1371/journal.pone.0181337)
Supplement: S1 File — (DOC) [file pone.0181337.s003.doc]

**S1 File**

List of the studied material and Accession Numbers of the Coleccion Aracnológica of the Museo Argentino de Ciencias Naturales “Bernardino Rivadavia” (MACN-Ar) of the newly sequenced specimens used in the species delimitation analysis.

***Ananteris balzanii*** Thorell, 1891. MACN-Ar 35701. Argentina, Formosa Province, Reserva Natural Formosa, (24º13’56.07’’S; 61º46’55.43’’W), III/2014; Ojanguren-Affilastro & Adilardi coll.

***Tityus argentinus*** Borelli 1899.MACN-Ar 35705. Argentina, Jujuy province, Calilegua National Park, Monolito, (23º40’52.3’’S; 64º54’05.1’’W), 1700asl, III/2013; Adilardi, Ojanguren-Affilastro, Rubio, Porta & Iuri coll.

***Tityus bahiensis*** (Perty 1833). MACN-Ar 35706.Argentina, Misiones Province, Saltos del Tabay, (26°59’59.34’’S; 55°10’41.62’’W), I/2012, Adilardi & Acuña coll.

***Tityus confluens*** Borelli 1899. MACN-Ar 35709. Argentina, Formosa Province, Reserva Natural Formosa, (24º13’56.07’’S; 61º46’55.43’’W), III/2014; Ojanguren-Affilastro & Adilardi coll.

***Tityus curupi* n. sp**. MACN-Ar 35723-35724. Argentina, Corrientes Province, General San Martín Department, Paraje Tres Cerros, Estancia Higuera-Cue, Cerro Chico, (29º06’48.85’’S; 56º55’05.81’’W); 22-24/II/205; Ojanguren-Affilastro, Adilardi, Piacentini & Ramírez Coll.

***Tityus curupi* n. sp**. MACN-Ar 35693-35694-35695-35696.Argentina, Corrientes Province, General San Martín Department, Paraje Tres Cerros, Estancia Higuera-Cue, Cerro Nazareno (29º06’34.59’’S; 56º55’55.27’’W); 22-24/II/205; Ojanguren-Affilastro, Adilardi, Piacentini & Ramírez Coll.

***Tityus paraguayensis*** Kraepelin 1895. MACN-Ar 35711. Argentina, Santa Fe Province, Laguna El Cristal, (30°01’13.23’’S; 60°06’27.88’’W); III/2014; Adilardi & Ojanguren-Affilastro coll.

***Tityus trivittatus*** Kraepelin 1898. MACN-Ar 35713. Argentina, Ciudad Autónoma de Buenos Aires, (34°35’39.39‘‘S; 58°24’41.58‘‘W); 26/X/2016; Adilardi & Ojanguren-Affilastro coll.

***Tityus uruguayensis*** Borelli 1901. MACN-Ar 35714-35715-35716-35717-35718-35719. Argentina, Entre Rios Province, El Palmar National Park, area arround La Calera, (Ruins of a limestone quarry), in the coast of the Uruguay River, (31°52’35.84‘‘S; 58°12’26.11‘‘W); 19/II/2015; Adilardi & Ojanguren-Affilastro coll.

***Zabius birabeni*** [Mello-Leitão](https://fr.wikipedia.org/wiki/Cândido_Firmino_de_Mello-Leitão), 1938. MACN-Ar 36499. Argentina, Río Negro Province, Sierra Grande (41°36’37.27’’S; 65°22’31.87’’W); X/2014; Piacentini, Brescovit, Santos & Magalhaes coll.

***Zabius fuscus*** Thorell 1893. MACN-Ar35676. Argentina, Córdoba Province, Dique El Cajón, (30°51’45.29’’S; 64°33’39.15’’W), 1000 m asl; II/2013; Adilardi, Ojanguren-Affilastro, Rubio, Porta & Iuri coll.

***Zabius* sp1**. MACN-Ar 36495. Argentina, Córdoba Province, Chancani Provincial Reserve (31°23’06.67’’S; 65°25’01.11’’W); II/2013; Mattoni coll.

***Zabius* sp2**. MACN-Ar 36496. Argentina, Córdoba Province, Salinas Grandes, (30°36’35.04’’S; 65°33’35.81’’W); II/2013; Mattoni Coll.
